# Identification and Expression of Inward-Rectifying Potassium Channel Subunits in *Plutella xylostella*

**DOI:** 10.3390/insects11080461

**Published:** 2020-07-22

**Authors:** Xiaoyi Lai, Jie Xu, Haihao Ma, Zheming Liu, Wei Zheng, Jia Liu, Hang Zhu, Yong Zhou, Xiaomao Zhou

**Affiliations:** 1 Long Ping Branch, Graduate School of Hunan University, Changsha 410125, China; laixiaoyi@hnu.edu.cn (X.L.); zhengweiaimm@126.com (W.Z.); 2 Institute of Agricultural Biotechnology, Hunan Academy of Agricultural Sciences, Changsha 410125, China; xujie957270@126.com (J.X.); liuzheming2007@sina.com (Z.L.); jialiuv@126.com (J.L.); zhyhhnl@163.com (H.Z.); qimiaobuchugan@126.com (Y.Z.)

**Keywords:** *Plutella xylostella*, Kir channel, phylogenetic analysis, real-time PCR

## Abstract

In insects, inward-rectifying potassium (Kir) channels regulate vital physiological functions, such as feeding behavior, silk secretion, renal excretion, and immune function. Therefore, they offer promising potential as targets for insecticides. Three types of Kir subunits have been identified in Diptera and Hemiptera, but the Kir subunits of Lepidoptera still remain unclear. This study identified five Kir subunit genes (*pxkir1*, *pxkir2*, *pxkir3A*, *pxkir3B*, and *pxkir4*) in the transcriptome of *Plutella xylostella*. Phylogenetic analysis identified *pxkir1*, *pxkir2*, *pxkir3A*, and *pxkir3B* as orthologous genes of *kir1*–*3* in other insects. Interestingly, *pxkir4* may be encoding a new class of Kir subunit in Lepidoptera that has not been reported to date. To identify further Kir channel subunits of *P. xylostella*, the gene expression profiles of five *pxkir* genes were studied by quantitative real-time PCR. These *pxkir* genes are expressed throughout the development of *P. xylostella*. *pxkir1* and *pxkir2* were highly expressed in thoraxes and legs, while *pxkir3* (3A and 3B) and *pxkir4* had high expression levels in the midgut and Malpighian tubules. This study identified the composition and distribution of Kir subunits in *P. xylostella* for the first time, and provides useful information for the further study of Kir channel subunits in Lepidoptera.

## 1. Introduction

Inwardly rectifying potassium (Kir) channels were first discovered in 1949 [1]. They facilitate the flow of potassium ions into a cell in response to the hyperpolarization of the cell membrane; however, when the membrane is depolarized, there is little to no extracellular flow of potassium ions [2,3,4]. In mammals, Kir channels play important roles in the regulation of the heartbeat, renal function, cell excitability, and insulin release [5,6]. Kir abnormalities can cause a series of diseases of the heart, kidney failure, and diseases of the nervous system [7,8,9]. Kir channels share the same basic topology: (1) Four subunits combine and form a canonical K^+^ pore-forming transmembrane domain (TMD) and a large cytoplasmic domain [10,11]. (2) An ion channel may be regulated by two gates: one formed by the transmembrane (TM) helices and one formed by the G-LOOP at the apex of the cytoplasmic domain [12,13].

Kir channels can be divided into three subtypes: the traditional Kir subtype, which does not require auxiliary subunits, the G protein-dependent GIRK subtype, and the KATP subtype that can be regulated by both intracellular ATP and sulfonylurea receptors (SURs) [11,13,14]. It has been reported that in mammals, Kir channels form a superfamily consisting of seven subfamilies (Kir1–7) [3]. However, few subunits have been identified in insects, and only three subclasses of Kir channel subunits (Kir1–3) were reported for Hemiptera and Diptera [15,16,17,18,19]. For example, three subunit genes (*kir1*, *kir2*, and *kir3*) were reported for *Drosophila melanogaster* [16,20,21] and *Nilaparvata lugens* [19]. Interestingly, *kir2* and *kir3* are duplicated in both *Aedes aegypti* and *Anopheles gambiae* [17,18,22], and *kir3* is absent in aphids [23]. Kir channel subunits in Lepidoptera have not been reported to date.

The life-stage and tissue-specific expressions of Kir channel subunits in *D. melanogaster*, *A. aegypti*, and *N. lugens* have been investigated. *kir1* is highly expressed during the pupal stage and in Malpighian tubules in adults, and the *kir1* is highest expressed in the ovarian tubule of *A. gambiae* [19,21,24,25]. *kir2* and *kir3* are also highly expressed in Malpighian tubules [15,19,20,22,26]. In *A. gambiae*, *kir2* has three subclasses and *kir3* has two subclasses [17,18,22]. Because of the lack of comprehensive and systematic research and reports, further work is needed to identify the life-stages and tissue-specific expressions of these genes.

In insects, Kir channels exert important physiological functions. RNA interference of the *agkir1* gene in *A. gambiae* has been shown to result in decreased egg production [24]. The small molecules VU590 and VU625 can inhibit Kir1 and Kir2 in mosquitoes, which can lead to renal failure [27,28]. Knockdown of *kir1* in *D. melanogaster* can deteriorate the function of the salivary gland and increase the time spent feeding [29]. The DmKir2 channel regulates the release of the bone morphogenetic protein Dpp in the developing wing of *Drosophila*, which is necessary for developmental signaling [30]. A recent report indicated that the insecticide flonicamid can act on the Kir channel of soybean aphids and *N. lugens*. Therefore, Kir channels have been identified as ideal insecticide targets [19].

*Plutella xylostella* is a destructive pest that causes enormous economic loss every year all over the world (4–5 billion US dollars per year) [31,32,33,34]. Studying the Kir channel will help to develop new strategies for the control of *P. xylostella*. This study analyzed the transcriptome *of P. xylostella*, screened the candidate Kir genes, and investigated their expression patterns, which will provide useful information for studying the Kir channels in *P. xylostella*.

## 2. Materials and Methods

### 2.1. Insects and Tissue Isolation

*P. xylostella* were cultured as previously described [35]. Tissue samples of *P. xylostella* at different life stages were collected as follows: each first instar sample consisted of 20 larvae, each second instar sample consisted of 10 larvae, each third instar sample consisted of five larvae, and each fourth instar sample consisted of three larvae. Moreover, each of the pre-pupal, female pupal, male pupal, female adult, and male adult samples contained three individuals, and each egg sample contained approximately 100 eggs. Larval tissue: The fourth instar larvae of *P. xylostella* were dissected under a stereomicroscope (ST70, SDPTOP, Shanghai, China) and different tissue samples were taken, including the head, epidermis, midgut, and Malpighian tubules. Each head sample contained tissues from 10 insects, each epidermal sample contained tissues from three insects, each midgut sample contained tissues from five insects, and each Malpighian tubule sample contained tissues from 10 insects. Adult tissues: female and male adults were dissected under a stereomicroscope and their heads, thoraxes, legs, wings, Malpighian tubules, midguts, ovarian tubules of female adults, and testes of male adults were extracted. Each tissue sample contained tissues from 15 adults (adult thorax tissue did not include legs or wings). Prior to tissue collection, each insect was first rinsed with 75% alcohol for 10 s, and then with sterile water, followed by sterile phosphate buffer saline (PBS, Solarbio, Beijing, China) for 15 s to remove surface residues. Three samples from each growth stage and tissue group were taken, to which 1 mL total RNA extraction reagent (Vazyme, Nanjing, China) was added. The samples were stored at −80 °C until later use.

### 2.2. RNA Isolation, cDNA Preparation, and TA Cloning

Kir subunits from *A. aegypti* (AaKir1:EAT39259, AaKir2A:EAT39257, AaKir2B:EAT39255, AaKir2B’:EAT34380, and AaKir3:EAT47212), *A. gambiae* (AgKir1:EAA01722, AgKir2A:EAA43222, AgKir2A’:EAA01723, AgKir2B:EAA43220, AgKir3A:EAL39730, and AgKir3B:AGAP028144), *D. melanogaster* (DmKir1:NP_001262861, DmKir2:ACL85253, and DmKir3:NP_001137835), and *N. lugens* (NlKir1:ASN63883, NlKir2:ASN63884, and NlKir3:ASN63885) were aligned. Conserved domains (protein sequences between TM1 helixes and G-LOOP) of each protein were used as markers to screen for *kir* genes in the two *P. xylostella* transcriptomes using Lepbase BLAST (http://blast.lepbase.org/, Accessed on 20 February 2017) [36]. Redundant sequences were deleted and five *pxkir* candidate genes (*pxkir1*, *pxkir2*, *pxkir3A*, *pxkir3B*, and *pxkir4*) were obtained. Specific primers were designed to amplify the full-length coding sequence (CDS) of *pxkir* genes (Table 1). Five *pxkir* genes were cloned and sequenced, and four distinct sequences were submitted to the GenBank database (*pxkir1*:MT274448, *pxkir2*:MN894569, *pxkir3A*:MN894570, and *pxkir3B*:MN894571). The *kir* genes of *Bombyx mori*, *Manduca sexta*, and *Danaus plexippus* were also investigated using the same method and the accession numbers of each gene are listed in Table 2.

Total RNA extraction and reverse transcription to cDNA were performed following previously described procedures [34]. The specific primers for PCR amplification of *kir* genes are listed in Table 1. To decrease the base mutations generated during PCR, the reaction was performed using Phanta Super-Fidelity DNA Polymerase. The reaction conditions are listed in Table A1. The PCR products were first separated and recovered via 1.0% agarose gel electrophoresis, followed by ligation to one of the following vectors using ligase. *pxkir* was ligated to the vector pTOPO (Aidlab Biotechnologies, Beijing, China). The ligated plasmid was then transformed into competent *Escherichia coli* DH5α cells and positive clones were screened on an LB agar plate (with 100 μg/mL Ampicillin) for plasmid extraction and sequence verification.

### 2.3. Sequences Alignment and Phylogenetic Analysis

The Kir channel subunit sequences of other insects were downloaded from GenBank and Clustal X was used for sequence alignment and to analyze the sequence similarity between PxKir subunits and Kir subunits of other species. The transmembrane regions of the protein were analyzed using the protein sequence analysis tool of ExPASy (TMHMM 2.0, http://www.cbs.dtu.dk/services/TMHMM-2.0/, accessed on 05 January 2017). The conserved protein domains were analyzed using the CD-search service (using the NCBI Conserved Domain Database CDD v3.18–55570 PSSMs, http://www.ncbi.nlm.nih.gov/Structure/cdd/wrpsb.cgi, accessed on 4 January 2017). Kir channel amino acid sequences from different species were collected and their evolutionary tree was analyzed using the neighbor-joining method based on the Poisson modified model in MEGA7 software [37].

### 2.4. Quantitative Real-Time PCR

Head and epidermal samples contained the majority of the central nervous system (CNS) of the organism. Both the midgut and Malpighian tubules are important for digestion and absorption of nutrients as well as for waste discharge. Thorax samples in adults are enriched with muscle tissue, and the ovarian tubules and testes are important for reproduction. Four tissues of larvae and eight tissues of adults were selected for tissue-specific expression analysis. Total RNA was extracted from different samples following the instruction manual of the manufacturer (Vazyme, Nanjing, China). A NanoDrop™ 1000 (Thermo, Wilmington, DE, USA) was used to quantify the extracted total RNA. A 1-μg RNA sample was used in the HiScript Q RT SuperMix for the qPCR (+gDNA wiper) reverse transcription kit. For cDNA synthesis, the total reaction volume was 20 μL. Synthesized cDNA samples were stored at −80 °C until further use. Quantitative analyses of *P. xylostella* cDNA samples at different life stages were performed using the FastStart Essential DNA Green Master kit (Roche, Basel, Switzerland). A 15-ng cDNA sample, which was reverse transcribed from total RNA, was used as a template for PCR, and Kir subunit genes were amplified using specific primers (see Table 1). *P. xylostella ribosomal protein S4* (*rpS4*, XM_011555372) was amplified and used as internal reference for the quantitative PCR analysis of different insect growth stages. *Ribosomal protein L8* (*rpL8*, NM_001305488) was amplified and used as internal reference for quantitative PCR analyses of different tissues [38,39]. *Elongation factor 1* (*ef1*, EF417849) was also amplified and used to verify the quantitative results (primers shown in Table 1). Three replicates were used per sample. The LightCycler^®^ 96 PCR instrument (Roche Molecular Systems, Inc., Basel, Switzerland) was used for real-time quantitative analysis. The two-step amplification program was conducted as follows: pre-denaturation at 95 °C for 30 s, followed by sample denaturation at 95 °C for 10 s and annealing at 59 °C for 30 s for 40 cycles. The fluorescence value was read at the end of each cycle. The results of three independent samples were analyzed and the expression differences between different samples were compared using the 2^−ΔΔCT^ method [40,41].

### 2.5. Data Analysis and Statistics

Because of the skewed and wide distribution of the data, gene expression values were log2 transformed, which enabled the application of parametric statistics. As the log of zero is undefined, one was added to all measurements to preserve the zeros in the derived data set [42]. All studies were replicated at least three times and the results were plotted as the means ± standard deviations (SD). One-way analyses of variance (ANOVA) were used, followed by the Tukey–Kramer post-hoc test using GraphPad Prism version 6.01 (GraphPad Software Inc., San Diego, CA, USA).

## 3. Results

### 3.1. Kir Channel Subunits in Lepidoptera

The genes of Kir channel subunits of four lepidopteran insects (*P. xylostella*, *B. mori*, *M. sexta*, and *D. plexippus*) were investigated and 12 partial or complete CDS of *kir* were identified in the transcriptome of *P. xylostella*. Furthermore, 19 sequences of *kir* were identified in the transcriptome of *B. mori*, as well as 13 *kir* sequences in both *M. sexta* and *D. plexippus*. After removal of redundant sequences, five *kir* genes were identified in both *P. xylostella* and *D. plexippus*, as well as six *kir* genes in both *B. mori* and *M. sexta* (Table 2).

**Figure 1:**
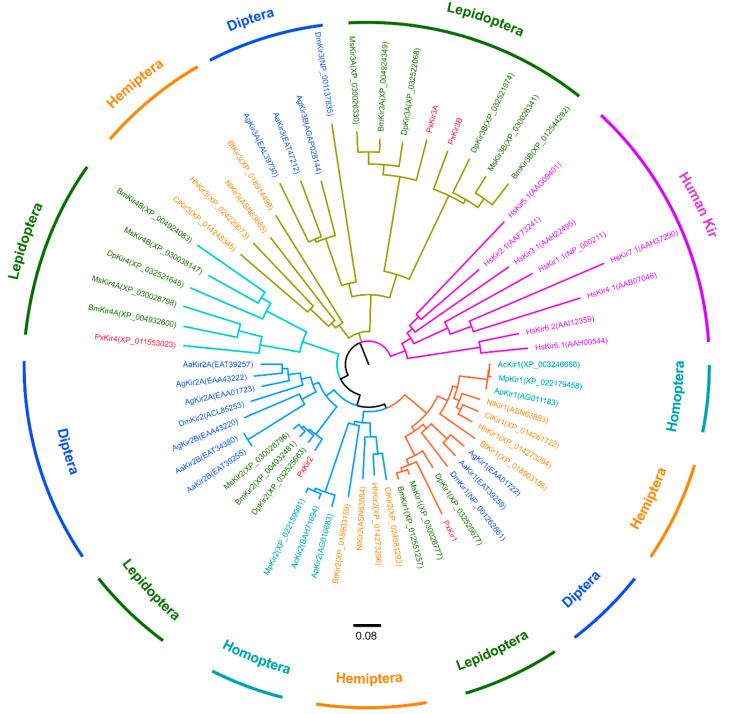
Phylogenetic analysis of PxKirs and various Kir subunits. Neighbor-joining tree showing the phylogenetic relationships of insect and human Kir channels (according to their amino-acid sequences). MEGA 7 was used to construct the tree (the neighbor-joining method was used; bootstrap test used 1000 replicates; model; amino: Poisson correction; gaps and treatment by pairwise deletion). The PxKir subunit is indicated with red color. GenBank accession numbers of Kir proteins are shown in parentheses. Abbreviations of species in alphabetical order: Aa: *Aedes aegypti*; Ac: *Acyrthosiphon pisum*; Ap: *Aphis glycines*; Ag: *Anopheles gambiae*; Bm: *Bombyx mori*; Bt: *Bemisia tabaci*; Cl: *Cimex lectularius*; Dm: *Drosophila melanogaster*; Dp: *Danaus plexippus*; Hh: *Halyomorpha halys*; Hs: *Homo sapiens*; Mp: *Myzus persicae*; Ms: *Manduca sexta*; Nl: *Nilaparvata lugens*; Px: *Plutella xylostella*.

To identify the subclasses of Lepidoptera candidate proteins, 40 previously reported Kir subunit sequences were downloaded from the GenBank database and a neighbor-joining method was used to construct a phylogenetic tree. These sequences included eight human-derived Kir proteins, 14 Kir sequences originating from Diptera (*A. aegypti*, *A. gambiae*, and *D. melanogaster*), six Kir sequences from Homoptera (*Acyrthosiphon pisum*, *Aphis glycines*, and *Myzus persicae*), and 12 Kir sequences from Hemiptera (*N. lugens*, *C. lectularius*, *B. tabaci*, and *H. hahalys*). The results indicated that insect Kir proteins and human Kir proteins form two independent evolutionary branches. The Kir proteins of Lepidoptera and other insects (i.e., the investigated Diptera and Hemiptera) were located on the same large branch of the phylogenetic tree (Figure 1).

The Kir1 subunits of Lepidoptera and Diptera are located on the same sub branch, while the Kir1 of Hemiptera and Homoptera are located on a different sub branch. Similarly, Kir2 and Kir3 in Lepidoptera, Diptera, Homoptera, and Hemiptera have the same evolutionary relationships with Kir1. Surprisingly, Kir3 from Lepidoptera have two subtypes (Kir3A and Kir3B) and compared with those of Diptera, both Kir3 subunits of Lepidoptera are located on the same sub branch. Moreover, two Kir4 subunits were identified in Lepidoptera. *B. mori* and *M. sexta* have Kir4A and Kir4B, but *P. xylostella* and *D. plexippus* only have one Kir4. In Lepidoptera, Kir4 was not located on the same branch as Kir1–3, suggesting that it may be a new subclass of the Kir channel in insects.

### 3.2. The pxkirs Clone

*P. xylostella* is an important vegetable pest and five Kir-subunit genes from *P. xylostella* were cloned and sequenced. The results showed that most *pxkirs* harbored alternative exons, and the *pxkirs* with the highest splicing frequency were selected for further research in this study. *pxkir1* has a full-length open reading frame (ORF) of 1665 bp, and the putative PxKir1 protein has 554 amino acids and a molecular weight (MW) of 63.5 kDa. The CDS of *pxkir2* has a length of 1566 bp, and the putative PxKir2 protein has 521 amino acids with a MW of 59.3 kDa. *pxkir3A* and *pxkir3B* are relatively similar in size. *pxkir3A* has a length of 1785 bp, and encodes 594 amino acids (MW: 67.2 kDa). *pxkir3B* has a sequence length of 1728 bp and encodes 575 amino acids (MW: 65.8 kDa). *pxkir4* is the shortest, with a full-length ORF of 1230 bp, and putative PxKir4 has 409 amino acids with an MW of 47.7 kDa.

Sequence homology analysis between Kir subunits in *P. xylostella* showed that the five putative PxKir subunits have less than 52% sequence similarity and the conservative domains (i.e., the sequences between TM1 and TM2) have less than 73% sequence similarity (Table 3). Specifically, the PxKir1 subunit had 50% similarity with PxKir2, PxKir2 had 51.9% similarity with PxKir4, PxKir3A had 40.8% similarity with PxKir3B, and other sequence similarities remained below 38%. This indicated that Kir subfamilies are significantly different from each other.

### 3.3. Sequence Analysis

**Figure 2:**
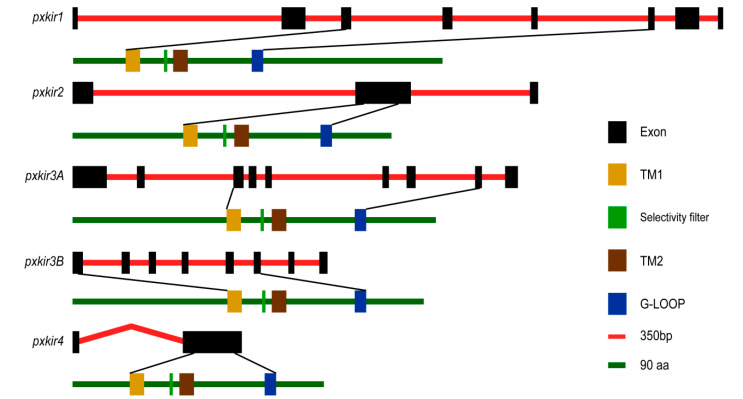
Genomic structures of *pxkir* and conservative domains of the deduced proteins. The exon−intron organization of *pxkir* was determined by sequence comparison between genomic sequence and cDNA sequence. The red line of every gene represents the original genomic scaffold sequences and the exons are indicated as black boxes. The green line indicates the deduced protein sequence. The locations encode the two transmembrane helices (TM1 and TM2), selectivity filters, and G-LOOP, which are marked by colored rectangles.

Genomic sequence analysis indicated that the *pxkir1* genomic sequence has a length of 12.1 kb and encodes eight exons. The *pxkir2* genomic sequence is 8.9 kb in length and contains three exons. The *pxkir3A* genomic sequence is 8.5 kb and contains nine exons. The *pxkir3B* genomic sequence is 4.8 kb and contains eight exons. The *pxkir4* is encoded by two exons and has an intron of unknown length (Figure 2). Transmembrane region prediction and CD-search analysis showed that the five deduced proteins all have the conservative domains of the Kir subunit, including the TM1, TM2, and G-LOOP domains. However, the position of conservative regions in the sequence is not consistent. The upstream sequence of TM1 in both PxKir1 and PxKir4 is shorter than other PxKir subunits, while the downstream sequence of the G-LOOP in PxKir1 is longer than that of other PxKir subunits (Figure 2).

Sequence alignments were performed between the PxKir subunits and orthologous in *D. melanogaster*, *A. aegypti*, and *N. lugens*. The results showed that: PxKir1 has a 52–67% sequence similarity and PxKir2 has a 61–77% sequence similarity with orthologous. PxKir3A and PxKir3B have only 32–39% sequence similarity with Kir3 in *D. melanogaster*, *A. aegypti*, and *N. lugens*, indicating that Kir3 showed significant polymorphism between different species. For PxKir4, 59–61% sequence similarity was found for Kir4A and 43–51% for Kir4B in Lepidoptera (Table A2).

**Figure 3:**
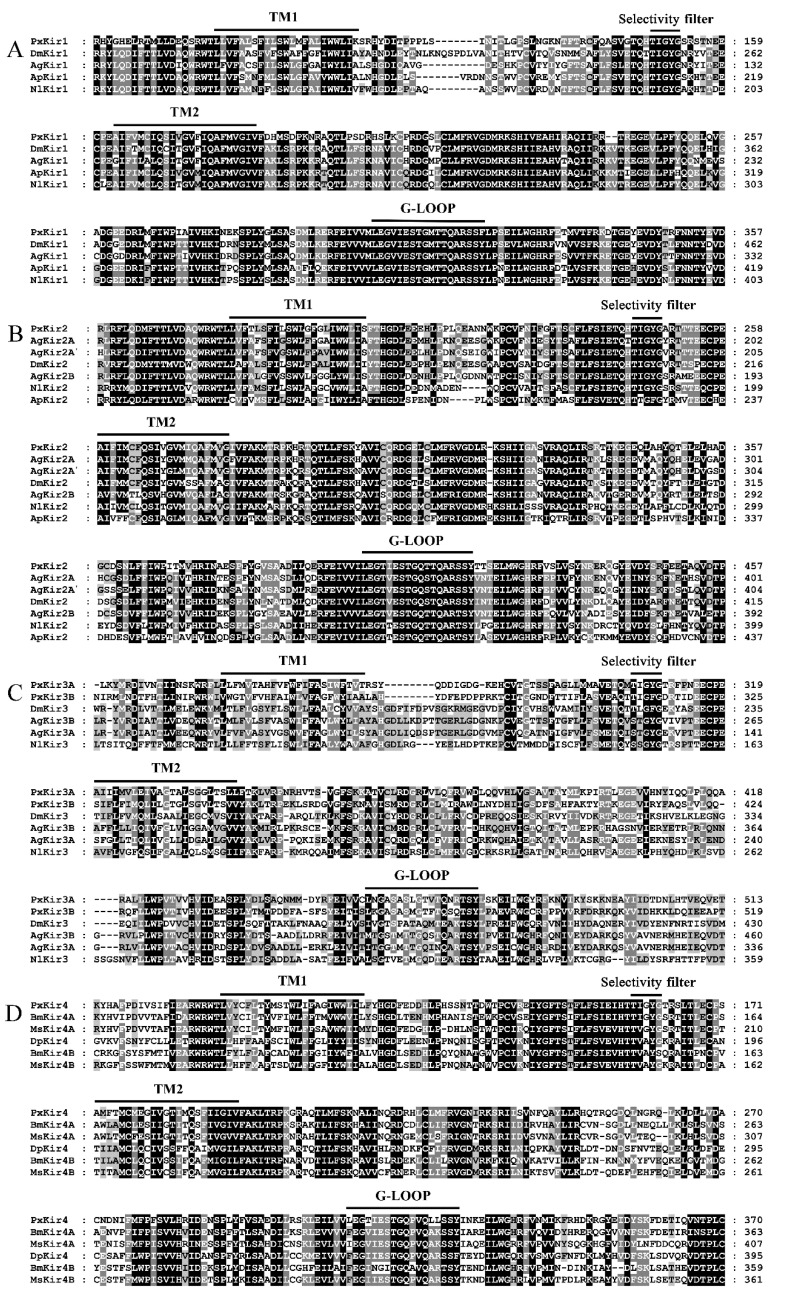
Multiple sequence alignment of putative PxKirs and other orthologous proteins in conservative domains. The respective positions are indicated on the right. Identical residues between orthologous sequences are shown as white characters against a black background, and conservative substitutions are shaded. The transmembrane helices (TM1 and TM2), the selectivity filter (SF), and the G-LOOP are highlighted with black lines. The conservative domains of Kir1 (A), Kir2 (B), Kir3 (C), and Kir4 (D) were aligned. GenBank accession numbers: *A. gambiae* (AgKir1, EAA01722; AgKir2A, EAA43222; AgKir2A’, EAA01723; AgKir2B, EAA43220; AgKir3A, EAL39730; and AgKir3B, AGAP028144); *A. glycines* (ApKir1, AG011183; and ApKir2, AG010683); *D. melanogaster* (DmKir1, NP_001262861; DmKir2, ACL85253; and DmKir3, NP_001137835); *N. lugens* (NlKir1, ASN63883; NlKir2, ASN63884; and NlKir3, ASN63885); *P. xylostella* (PxKir1–3, in this study; and PxKir4, XP_011553023); *B. mori* (BmKir4A, XP_004932600; and BmKir4B, XP_012544061); *M. sexta* (MsKir4A, XP_030028798; and MsKir4B, XP_030038147); and *D. plexippus* (DpKir4, XP_032521645).

Sequence alignments also showed that all PxKir subunits contained two TM helices (TM1 and TM2) and a K^+^ selectivity filter (containing a “TIGYG” motif between both TM helices). These five candidate PxKir subunits all presented the typical conservative domain features of Kir subunits and should be able to form functional potassium channels (Figure 3). Interestingly, the selectivity filters of Kir4A and Kir4B were significantly different; the sequence was “TI(V)GVG” for Kir4A and “TVAYG(S)” for Kir4B.

### 3.4. Developmental and Tissue Distribution of kirs in P. xylostella

**Figure 4:**
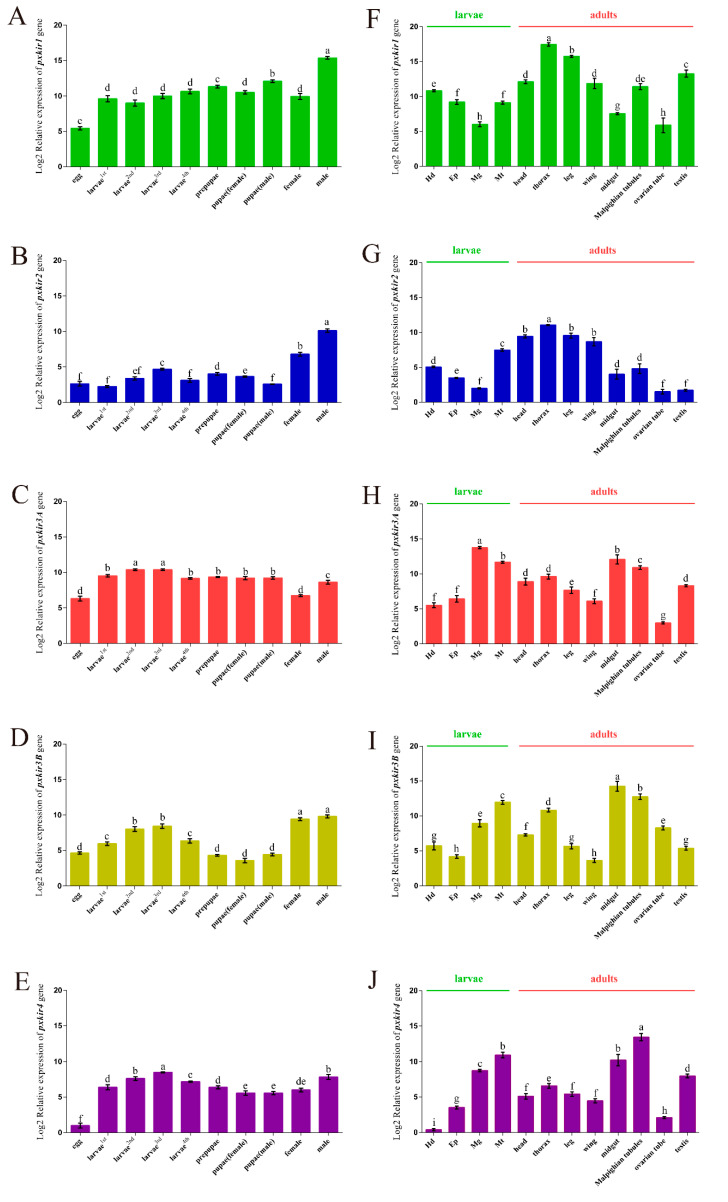
Log2 relative mRNA expression levels of *pxkirs* at different developmental stages (**A**–**E**) and in different tissues (**F**–**J**). Different developmental stages of *P. xylostella*, including eggs, 1st to 4th instar larvae, prepupae, pupae (female and male), and adults (female and male). The expression level of *pxkir4* during the egg period was defined as a benchmark for statistical analysis, using *rpS4* as the reference gene. Different tissues come from larvae (fourth instar) and adults. Tissue abbreviations: head (Hd), epidermal samples (Ep), Malpighian tubules (Mt), and midgut (Mg). The expression level of the *pxkir4* during the egg period was defined as a benchmark for statistical analysis, by using *rpL8* as a reference gene. Different lower-case letters above the bars indicate significant differences. The data are presented as means ± SD (N > 3).

The five *pxkirs* expression profiles were investigated by qRT-PCR. The expression levels of *pxkir1* were relatively high throughout all life stages, and the highest mRNA level was found in male adults (the expression was about 1000-fold of the expression level of the *pxkir1* in eggs, 95% confidence interval (CI): 953 to 1207-fold, Figure 4A). During the adult stage in different tissues, the highest expression level was found in the thorax, followed by legs and testis, and the *pxkir1* mRNA levels were lower in the midguts of larva and the ovarian tubules of female adult. The expression in the thorax was significantly higher than in other tissues, which was more than 2500 times that of the lowest *pxkir1* expression in the ovarian tubule (95% CI: 1770 to 4096-fold, Figure 4F).

The *pxkir2* mRNA levels were low at the larval and pupae stages, and the highest expression level was found in male adults (approximately 500-fold of the level found in eggs, 95% CI: 425 to 603-fold, Figure 4B). Similar to *pxkir1*, the highest expression of *pxkir2* at the adult stage was in locomotor tissues, and in the thorax, it was approximately 700 times that of the ovarian tubule (95% CI: 619 to 1531-fold, Figure 4G). The expression levels of *pxkir1* and *pxkir2* differed in reproductive organs. *pxkir1* was highly expressed in testes and ovarian tubes (Figure 4F), but *pxkir2* showed low expression in both tissues (Figure 4G).

The *pxkir3A* was also highly expressed in the larval stage, like *pxkir1*, but it showed significantly lower expression at the pupae and adult stages (Figure 4C). The expression of the *pxkir3B* was lower during the pupal stage than that of *pxkir3A* (Figure 4D). *pxkir3A* was highly expressed in midguts and Malpighian tubules (more than 6000 times in midgut of the larvae stage than that in the ovarian tube, 95% CI: 6019 to 6931-fold, nearly 1500 times in Malpighian tubules of the adult stage than that in the ovarian tube, 95% CI: 1181 to 2093-fold, Figure 4H). *pxkir3B* was also highly expressed in the midgut and in Malpighian tubules (Figure 4I). *pxkir3A* was highly expressed in testes and *pxkir3B* was highly expressed in ovarian tubules.

The expression of *pxkir4* was similar to that of *pxkir3B,* and *pxkir4* was almost not expressed in eggs and had low expression in female adults (Figure 4E). Tissue-specific expressions showed that the *pxkir4* expression profile was similar to that of *pxkir3*, and the highest expression was found in Malpighian tubules, followed by the midgut (Figure 4J). Unlike *pxkir3*, *pxkir4* was not expressed in the head of larvae and showed only low expression in the head of adults.

## 4. Discussion

Kir channels regulate important physiological functions in insects, such as egg production [24], renal function [27,28,43], salivary gland function and feeding [29], developmental signaling [30], and the antiviral immune response [44]. Kir channels have been proposed to be a target of the insecticide flonicamid [19]. Via patch clamp technology, the electrophysiological characteristics of several insect Kir channels have been measured, which provided an efficient tool for the functional research of genes and for Kir-targeting insecticide development [17,22,24,25,43]. At present, most research of the Kir channel focused on Diptera and Hemiptera, and the reports on Lepidoptera are few. Since many important economic insects (e.g., morpho butterfly and silkworm) and agricultural pests (e.g., bollworm, cotton leafworm, cabbage worm, and the diamondback moth) belong to Lepidoptera, it is necessary to investigate the classification and function of Kir subunits in Lepidoptera.

In this study, five *kirs* were identified in both *P. xylostella* and *D. plexippus*, and six *kirs* were identified in both *B. mori* and *D. plexippus*. The *kir3* and *kir4* in Lepidoptera are diverse, leading to A and B subtypes. The same diversity of Kir2 was reported in mosquitoes [22,45]. Only one *kir4* (*kir4A*) was identified in *P. xylostella*. The *bmkir4B* and *mskir4B* sequences were used for BLAST analysis of the transcriptome of *P. xylostella*, which did not yield meaningful results. Maybe, the Kir4B was absent because of functional redundancy in *P. xylostella*. Similarly, the Kir3 subunit was absent from the transcriptome and genome of *A. glycines* [23] and two Kir channels (Irk1 and Irk2) play redundant roles in the function of the renal tubules of *Drosophila* [25]. Two Kir subunits were reported in *A. glycines* [23], three Kir subunits were reported in *D. melanogaster* and *N. lugens* [19,25], and four to five Kir subunits were reported in mosquitoes [18,24]. The composition of Kir subunits in different species is quite different; whether the same Kir subtypes that have been identified by phylogenetic analysis have the same function requires further study.

The expression and function of Kir subunits in *D. melanogaster, A. aegypti*, and *N. lugens* have been reported: Kir subunits are expressed in the insects’ Malpighian tubules [17,22,25,26], CNS [46], muscle [30], salivary glands [19,29], and reproductive organs [24], where they regulate the insects’ renal function, heart function, patterning, salivary gland function, and virus resistance. This study also investigated the expression of Kir subunits in the CNS (head), digestion and metabolite excretion organs (midgut and Malpighian tubules), locomotive organs (thorax, legs, wings), and reproductive organs (ovarian tubule and testis) of *P. xylostella*. The *pxkirs* (except *pxkir2*) were highly expressed in the Malpighian tubules, *pxkir*1–3 was enriched in the head, *pxkir1* and *pxkir2* were highly expressed in the thorax and legs, *pxkir3* and *pxkir4* were highly expressed in the midgut, *pxkir2* was highly expressed in the wings, *pxkir3B* was highly expressed in the ovarian tubule, and *pxkir1*, *pxkir3A*, and *pxkir4* were highly expressed in the testes (Figure 4). These data suggested that Kirs may play important roles in the regulation of renal function, gland secretion, locomotion and reproduction of *P. xylostella*. In particular, Kir3 and Kir4 were highly expressed in Malpighian tubules, which may be potential insecticide targets like AeKir1–2 [15,24,43]. In any case, these data provide useful information for further study of the physiological functions of different Kir subunits in *P. xylostella*.

Unexpectedly, significant differences of expression were found between *pxkirs* and homologous genes. For example, comparing the expression levels of *kir1* in different development stages showed that *kir1* was highly expressed in the pupa and adult stages of *D. melanogaster* [20] and *A. aegypti* [18], but was only highly expressed in the pupa stage in *A. gambiae* [24]. This study showed that *pxkir1* was highly expressed throughout all growth stages, and the highest level of *pxkir1* mRNA was found in male adults (Figure 4A). Comparing the tissue expression differences of *kir1* showed that *kir1* was highly expressed in the ovarian tubule of *A. aegypti* and *A. gambiae*, and was less expressed in the midgut and fat body [18,24]. However, the expression levels of *kir1* in the midgut of *C. lectularius* [47] and the fat body of *N. lugens* [19] were all higher than those in the Malpighian tubules. In this study, *pxkir1* showed low expression in the ovarian tubule and high expression in the testes (Figure 4F). Moreover, it was difficult to confirm the common expression pattern of Kir2 and Kir3 in different insects because of the gene diversity and alternative splicing of Kir2 [48,49]. In this study, three kinds of alternative splicing were identified for *pxkir1* (data not shown). It remains unclear whether the diversity and alternative splicing of *kir* affect the function of Kir subunits in *P. xylostella*.

The PxKir4 subunit showed high sequence similarity with PxKir2 (59.2%) and both genes showed a similar extron–intron structure. *pxkir4* had a higher expression in the larval midgut and Malpighian tubules (Figure 4J), and a lower expression in the head and eggs (Figure 4E), which is similar to *kir2* in other insects [15,20,49]. This suggests that *pxkir4* is more closely related to *kir2*. This study showed that the expression profile of *pxkir4* was closer to that of *pxkir3*, and because of a lack of support by evolutionary analysis and functional analysis, Kir4 was identified as a new type of Kir subunit in Lepidoptera.

## 5. Conclusions

In this study, five candidate Kir genes (*pxkir1*, *pxkir2*, *pxkir3A*, *pxkir3B*, and *pxkir4*) from *P. xylostella* were cloned for the first time. Sequence analysis showed that all cloned *pxkir* genes could encode the common conservative domains of the Kir subunit. Phylogenetic analysis suggested that Kir4 in Lepidoptera may be a new subtype of the Kir subunit. The temporal and spatial expressions of the *kir* genes in *P. xylostella* were also characterized. These results elucidate the composition and distribution of Kir subunits in *P. xylostella* and provide useful information for the study of Kir channels in *P. xylostella*.

## Figures and Tables

**Table 1:** Primers used for clone and expression analysis of genes in *Plutella xylostella*.

Name	Primer Sequences (5′-3′)	Gene	Site	Purpose
PxKir1_F	ATGAAGAGATGTCGGCCATGAAGAGAT	*pxkir1*	−8	DNA cloning
PxKir1_R	TACACTTCTGCGTCTGGTGCTGACG		1861	
PxKir2_F	CTTTCGTATTTAGTATCTATGGTT	*pxkir2*	−92	
PxKir2_R	AATGAGATAGTTCGTAAAGTTGCT		1830	
PxKir3A_F	TAAGTTGGACACATCATTGAGCTT	*pxkir3A*	−135	
PxKir3A_R	GAAAATCAGTTTCCTACGTTGGATAGTT		1997	
PxKir3B_F	CTGCTGTAGGCTGATGATGGTGAT	*pxkir3B*	−123	
PxKir3B_R	AAGTCAAGAATCGTTTTGTTATGTG		1890	
PxKir4_F	TTTACCGGCATGGCATTCTATTACG	*pxkir4*	−9	
PxKir4_R	GAAAATGAAGGTAGCAACTTTACAAATACA		1375	
PxKir1-qF	GAGGTGCTGCCGTTCTAC	*pxkir1*	703	Real-time Quantitative PCR
PxKir1-qR	TCGACTCGATCACTCCCT		895	
PxKir2-qF	ATTGGACAGTTCACGATACATAGGAA	*pxkir2*	336	
PxKir2-qR	CCGAGCCAGGAGAGGATGAAG		575	
PxKir3A-qF	GGGGGTACCGCTTCAAGAATGTTAT	*pxkir3A*	1441	
PxKir3A-qR	GGAGGAACCCTGTGTGACTGAG		1626	
PxKir3B-qF	ACAAGGCTGGCTATGGCGACTA	*pxkir3B*	610	
PxKir3B-qR	GATGTACCAGAAACCAGCGAAC		822	
PxKir4-qF	TATCATCCAGGAAGTGGCTAAGGCT	*pxkir4*	62	
PxKir4-qR	GTCGGGAAACGCATGGTACTTGTGT		234	
RPL8-qF	CGGTCGTGCCTACCACAAATACA	*rpL8*	559	
RPL8-qR	CGTGAGGATGCTCCACAGGGT		648	
RPS4-qF	ATGGATGTTGTGTCGATTGAAAAGA	*rpS4*	335	
RPS4-qR	GAGTGATGCGGTGGATGGTGA		423	
EF1-qF	GCCTCCCTACAGCGAATC	*ef1*	374	
EF1-qR	CCTTGAACCAGGGCATCT		535	

**Table 2:** The *kir* genes of *P. xylostella*, *B. mori*, *M. sexta*, and *D. plexippus* in GenBank.

Gene	*P. xylostella*	*B. mori*	*M. sexta*	*D. plexippus*
*kir1*	g814.t1 * (MT274448)	XM_012695801.2	XM_030172912	XM_032669786
*kir2*	XM_011554745 (MN894569)	XM_004932424.3	XM_030172936	XM_032669772
*kir3A*	XM_011568587 (MN894570)	XM_004924292.3	XM_030170470	XM_032666177
*kir3B*	XM_011564409 (MN894571)	XM_021346768.1	XM_030170480	AGBW02010886
*kir4A*	XM_011554721	XM_004932543.3	XM_030172938	-
*kir4B*	-	XM_012688607.2	XM_030182287	XM_032665754

* *kir1* in Lepbase (http://ensembl.lepbase.org/Plutella_xylostella_pacbiov1/Info/Index accessend on 20 February 2017). The accession numbers of the *pxkir* gene in this study are shown in parentheses.

**Table 3:** Amino acid sequence similarity between *Plutella xylostella* Kir subunits.

Channel	PxKir1 (%)	PxKir2 (%)	PxKir3A (%)	PxKir3B (%)	PxKir4 (%)
PxKir1	100	50.0 (72.0)	27.0 (41.7)	37.7 (45.8)	36.0 (47.3)
PxKir2		100	32.1 (72.7)	31.9 (62.1)	51.9 (59.2)
PxKir3A			100	40.8 (52.4)	35.3 (38.0)
PxKir3B				100	34.4 (43.1)
PxKir4					100

Note: The percentages of conservatively substituted amino acids (in parenthesis) between all five PxKir subunits were calculated via pairwise alignments of the complete amino acid sequences.

## Appendix A

**Table A1:** Gene amplification procedures of *kir* in *Plutella xylostella.*

Cycle Steps	*pxkir1*	*pxkir2*	*pxkir3a*	*pxkir3b*	*pxkir4*	Cycles
Initial denaturation	95 °C 3 min	95 °C 3 min	95 °C 3 min	95 °C 3 min	95 °C 3 min	1
Denaturation	95 °C 15 s	95 °C 15 s	95 °C 15 s	95 °C 15 s	95 °C 15 s	32
Annealing	60 °C 15 s	50 °C 15 s	62 °C 15 s	58 °C 15 s	60 °C 15 s	
Extension	72 °C 120 s	72 °C 90 s	72 °C 100 s	72 °C 100 s	72 °C 90 s	
Final elongation	72 °C 5 min	72 °C 5 min	72 °C 5 min	72 °C 5 min	72 °C 5 min	1

**Table A2:** Amino acid sequence similarity of Kirs between *Plutella xylostella* and other insects.

Kir of *P. xylostella*	Kir of Other Insects	Similarity (%)
PxKir1	AaKir1	52
	AgKir1	54
	DmKir1	52
	NlKir1	67
	ApKir1	61
PxKir2	AaKir2A	77
	AaKir2B	64
	AaKir2B’	64
	AgKir2A	74
	AgKir2A’	67
	AgKir2B	61
	DmKir2	69
	NlKir2	67
	ApKir2	55
PxKir3A	AaKir3	33
	AgKir3A	39
	DmKir3	34
	NlKir3	32
PxKir3B	AaKir3	39
	AgKir3A	38
	DmKir3	38
	NlKir3	36
PxKir4	BmKir4A	61
	BmKir4B	47
	MsKir4A	59
	MsKir4B	51
	DpKir4	47

## References

[1] Minor D.L., Masseling S.J., Jan Y.N., Jan L.Y. (1999). Transmembrane structure of an inwardly rectifying potassium channel. Cell.

[2] Lu Z., Klem A.M., Ramu Y. (2001). Ion conduction pore is conserved among potassium channels. Nature.

[3] Hibino H., Inanobe A., Furutani K., Murakami S., Findlay I., Kurachi Y. (2010). Inwardly rectifying potassium channels: Their structure, function, and physiological roles. Physiol. Rev..

[4] Gonzalez C., Baez-Nieto D., Valencia I., Oyarzun I., Rojas P., Naranjo D., Latorre R. (2012). K^+^ channels: Function-structural overview. Compr. Physiol..

[5] Abraham M.R., Jahangir A., Alekseev A.E., Terzic A. (1999). Channelopathies of inwardly rectifying potassium channels. FASEB J..

[6] Pattnaik B.R., Asuma M.P., Spott R., Pillers D.A. (2012). Genetic defects in the hotspot of inwardly rectifying K^+^ (Kir) channels and their metabolic consequences: A review. Mol. Genet. Metab..

[7] Mayfield J., Blednov Y.A., Harris R.A. (2015). Behavioral and Genetic Evidence for GIRK Channels in the CNS: Role in Physiology, Pathophysiology, and Drug Addiction. Int. Rev. Neurobiol..

[8] Sacco S., Giuliano S., Sacconi S., Desnuelle C., Barhanin J., Amri E.Z., Bendahhou S. (2015). The inward rectifier potassium channel Kir2.1 is required for osteoblastogenesis. Hum. Mol. Genet..

[9] Tinker A., Aziz Q., Li Y.W., Specterman M. (2018). ATP-Sensitive Potassium Channels and Their Physiological and Pathophysiological Roles. Compr. Physiol..

[10] Clarke O.B., Caputo A.T., Hill A.P., Vandenberg J.I., Smith B.J., Gulbis J.M. (2010). Domain reorientation and rotation of an intracellular assembly regulate conduction in Kir potassium channels. Cell.

[11] Li N.N., Wu J.X., Ding D., Cheng J.X., Gao N., Chen L. (2017). Structure of a Pancreatic ATP-Sensitive Potassium Channel. Cell.

[12] Tao X., Avalos J.L., Chen J.Y., MacKinnon R. (2009). Crystal structure of the eukaryotic strong inward-rectifier K^+^ channel Kir2.2 at 3.1 A resolution. Science.

[13] Hansen S.B., Tao X., MacKinnon R. (2011). Structural basis of PIP2 activation of the classical inward rectifier K^+^ channel Kir2.2. Nature.

[14] Nishida M., MacKinnon R. (2002). Structural basis of inward rectification: Cytoplasmic pore of the G protein-gated inward rectifier GIRK1 at 1.8 A resolution. Cell.

[15] Rouhier M.F., Raphemot R., Denton J.S., Piermarini P.M. (2014). Pharmacological validation of an inward-rectifier potassium (Kir) channel as an insecticide target in the yellow fever mosquito *Aedes aegypti*. PLoS ONE.

[16] Reale V., Chatwin H.M., Evans P.D. (2004). The activation of G-protein gated inwardly rectifying K^+^ channels by a cloned *Drosophila melanogaster* neuropeptide F-like receptor. Eur. J. Neurosci..

[17] Piermarini P.M., Dunemann S.M., Rouhier M.F., Calkins T.L., Raphemot R., Denton J.S., Hine R.M., Beyenbach K.W. (2015). Localization and role of inward rectifier K^+^ channels in Malpighian tubules of the yellow fever mosquito *Aedes aegypti*. Insect Biochem. Mol. Biol..

[18] Yang Z.X., Statler B.M., Calkins T.L., Alfaro E., Esquivel C.J., Rouhier M.F., Denton J.S., Piermarini P.M. (2017). Dynamic expression of genes encoding subunits of inward rectifier potassium (Kir) channels in the yellow fever mosquito *Aedes aegypti*. Comp. Biochem. Physiol. B Biochem. Mol. Biol..

[19] Ren M.M., Niu J.G., Hu B., Wei Q., Zheng C., Tian X.R., Gao C.F., He B.J., Dong K., Su J.Y. (2018). Block of Kir channels by flonicamid disrupts salivary and renal excretion of insect pests. Insect Biochem. Mol. Biol..

[20] Doring F., Wischmeyer E., Kuhnlein R.P., Jackle H., Karschin A. (2002). Inwardly rectifying K^+^ (Kir) channels in *Drosophila*. A crucial role of cellular milieu factors Kir channel function. J. Biol. Chem..

[21] MacLean S.J., Andrews B.C., Verheyen E.M. (2002). Characterization of Dir: A putative potassium inward rectifying channel in *Drosophila*. Mech. Dev..

[22] Piermarini P.M., Rouhier M.F., Schepel M., Kosse C., Beyenbach K.W. (2013). Cloning and functional characterization of inward-rectifying potassium (Kir) channels from Malpighian tubules of the mosquito *Aedes aegypti*. Insect Biochem. Mol. Biol..

[23] Piermarini P.M., Inocente E.A., Acosta N., Hopkins C.R., Denton J.S., Michel A.P. (2018). Inward rectifier potassium (Kir) channels in the soybean aphid *Aphis glycines*: Functional characterization, pharmacology, and toxicology. J. Insect Physiol..

[24] Raphemot R., Estevez-Lao T.Y., Rouhier M.F., Piermarini P.M., Denton J.S., Hillyer J.F. (2014). Molecular and functional characterization of *Anopheles gambiae* inward rectifier potassium (Kir1) channels: A novel role in egg production. Insect Biochem. Mol. Biol..

[25] Wu Y.P., Baum M., Huang C.L., Rodan A.R. (2015). Two inwardly rectifying potassium channels, Irk1 and Irk2, play redundant roles in *Drosophila* renal tubule function. Am. J. Physiol. Regul. Integr. Comp. Physiol..

[26] Piermarini P.M., Esquivel C.J., Denton J.S. (2017). Malpighian Tubules as Novel Targets for Mosquito Control. Int. J. Environ. Res. Public Health.

[27] Lewis L.M., Bhave G., Chauder B.A., Banerjee S., Lornsen K.A., Redha R., Fallen K., Lindsley C.W., Weaver C.D., Denton J.S. (2009). High-throughput screening reveals a small-molecule inhibitor of the renal outer medullary potassium channel and Kir7.1. Mol. Pharmacol..

[28] Kharade S.V., Sheehan J.H., Figueroa E.E., Meiler J., Denton J.S. (2017). Pore Polarity and Charge Determine Differential Block of Kir1.1 and Kir7.1 Potassium Channels by Small-Molecule Inhibitor VU590. Mol. Pharmacol..

[29] Swale D.R., Li Z.L., Guerrero F., Perez De Leon A.A., Foil L.D. (2017). Role of inward rectifier potassium channels in salivary gland function and sugar feeding of the fruit fly, *Drosophila melanogaster*. Pestic. Biochem. Physiol..

[30] Dahal G.R., Pradhan S.J., Bates E.A. (2017). Inwardly rectifying potassium channels influence *Drosophila* wing morphogenesis by regulating Dpp release. Development.

[31] Fenner K., Canonica S., Wackett L.P., Elsner M. (2013). Evaluating pesticide degradation in the environment: Blind spots and emerging opportunities. Science.

[32] Zhang S.Z., Zhang X.L., Shen J., Li D.Y., Wan H., You H., Li J.H. (2017). Cross-resistance and biochemical mechanisms of resistance to indoxacarb in the diamondback moth, *Plutella xylostella*. Pestic. Biochem. Physiol..

[33] Yin C.Y., Wang R., Luo C., Zhao K., Wu Q.Y., Wang Z.Y., Yang G.F. (2019). Monitoring, Cross-Resistance, Inheritance, and Synergism of *Plutella xylostella* (Lepidoptera: Plutellidae) Resistance to Pyridalyl in China. J. Econ. Entomol..

[34] Xu J., Wang Z.Y., Wang Y.F., Ma H.H., Zhu H., Liu J., Zhou Y., Deng X.L., Zhou X.M. (2020). ABCC2 participates in the resistance of *Plutella xylostella* to chemical insecticides. Pestic. Biochem. Physiol..

[35] Ma H.H., Huang Q.T., Lai X.Y., Liu J., Zhu H., Zhou Y., Deng X.L., Zhou X.M. (2019). Pharmacological Properties of the Type 1 Tyramine Receptor in the Diamondback Moth, *Plutella xylostella*. Int. J. Mol. Sci..

[36] Zhou J.L., Guo Z.J., Kang S., Qin J.Y., Gong L.J., Sun D., Guo L., Zhu L.H., Bai Y., Zhang Z.Z. (2020). Reduced expression of the P-glycoprotein gene PxABCB1 is linked to resistance to *Bacillus thuringiensis* Cry1Ac toxin in *Plutella xylostella* (L.). Pest Manag. Sci..

[37] Huang Q.T., Ma H.H., Deng X.L., Zhu H., Liu J., Zhou Y., Zhou X.M. (2018). Pharmacological characterization of a beta-adrenergic-like octopamine receptor in *Plutella xylostella*. Arch. Insect Biochem. Physiol..

[38] Fu W., Xie W., Zhang Z., Wang S.L., Wu Q.J., Liu Y., Zhou X., Zhou X.M., Zhang Y.J. (2013). Exploring valid reference genes for quantitative real-time PCR analysis in *Plutella xylostella* (Lepidoptera: Plutellidae). Int. J. Biol. Sci..

[39] You Y.C., Xie M., Vasseur L., You M.S. (2018). Selecting and validating reference genes for quantitative real-time PCR in *Plutella xylostella* (L.). Genome.

[40] Hua J.F., Zhang S., Cui J.J., Wang D.J., Wang C.Y., Luo J.Y., Lv L.M., Ma Y. (2013). Functional characterizations of one odorant binding protein and three chemosensory proteins from *Apolygus lucorum* (Meyer-Dur) (Hemiptera: Miridae) legs. J. Insect Physiol..

[41] Livak K.J., Schmittgen T.D. (2001). Analysis of relative gene expression data using real-time quantitative PCR and the 2(-Delta Delta C(T)) Method. J. Methods.

[42] Wilkinson R., Wang X.J., Kassianos A.J., Zuryn S., Roper K.E., Osborne A., Sampangi S., Francis L., Raghunath V., Healy H. (2014). Laser capture microdissection and multiplex-tandem PCR analysis of proximal tubular epithelial cell signaling in human kidney disease. PLoS ONE.

[43] Raphemot R., Rouhier M.F., Hopkins C.R., Gogliotti R.D., Lovell K.M., Hine R.M., Ghosalkar D., Longo A., Beyenbach K.W., Denton J.S. (2013). Eliciting renal failure in mosquitoes with a small-molecule inhibitor of inward-rectifying potassium channels. PLoS ONE.

[44] O’Neal S.T., Swale D.R., Anderson T.D. (2017). ATP-sensitive inwardly rectifying potassium channel regulation of viral infections in honeybees. Sci. Rep..

[45] Raphemot R., Rouhier M.F., Swale D.R., Days E., Weaver C.D., Lovell K.M., Konkel L.C., Engers D.W., Bollinger S.R., Hopkins C. (2014). Discovery and characterization of a potent and selective inhibitor of *Aedes aegypti* inward rectifier potassium channels. PLoS ONE.

[46] Chen R., Swale D.R. (2018). Inwardly rectifying potassium (Kir) channels represent acritical ion conductance pathway in the nervous systems of insects. Sci. Rep..

[47] Mamidala P., Mittapelly P., Jones S.C., Piermarini P.M., Mittapalli O. (2013). Molecular characterization of genes encoding inward rectifier potassium (Kir) channels in the bed bug (*Cimex lectularius*). Comp. Biochem. Physiol. Part B.

[48] Rouhier M.F., Piermarini P.M. (2014). Identification of life-stage and tissue-specific splice variants of an inward rectifying potassium (Kir) channel in the yellow fever mosquito Aedes aegypti. Insect Biochem. Mol. Biol..

[49] Evans J.M., Allan A.K., Davies S.A., Dow J.A. (2005). Sulphonylurea sensitivity and enriched expression implicate inward rectifier K^+^ channels in *Drosophila melanogaster* renal function. J. Exp. Biol..

